# Traumatic anterior hip dislocation with associated bilateral femoral fractures in a child: a case report and review of the literature

**DOI:** 10.11604/pamj.2019.32.88.17497

**Published:** 2019-02-25

**Authors:** Zongbing Cao, Dong Zhu, Chen Li, Yan-Hui Li, Lei Tan

**Affiliations:** 1 Departments of Orthopedic Trauma, The First Hospital of Jilin University, Changchun, China; 2 Cardiology and Echocardiography, The First Hospital of Jilin University, Changchun, China

**Keywords:** Anterior dislocation, hip, femoral fracture, child

## Abstract

Traumatic anterior hip dislocation is rare, because the hip joint is a highly stable joint. It is extremely rare for the anterior hip dislocation with combined bilateral femoral fracture in children. We present a case of 7-year-old boy with traumatic anterior hip dislocation with associated bilateral femoral fractures. Radiographic examination showed the right femoral head was dislocated anteroinferiorly. The ipsilateral femoral shaft showed a transverse femoral shaft fracture and proximal and distal femoral bifocal fractures of the contralateral femur. The dislocation of the right hip was reduced 10 hours after the injury in local hospital. One week later, the right femoral shaft fracture and left proximal femoral fracture were open reduced and internally fixed with plate and screws and the distal left femoral fracture was closed reduced and fixed with Kirschner wires. Postoperatively, active hip flexion and extension to recover hip and knee movement were then permitted but without weight bearing for 3 months. Radiographs at 3 months, at 6 months showed the fractures healed well and hardware were removed respectively. However, irregularities of the femoral head meaned avascular necrosis of the femoral head. This case stresses the importance of a rapid evaluation and treatment for the dislocation of the hip, providing a stable reduction and a firm internal fixation of the associated fractures.

## Introduction

Traumatic hip joint dislocation is a rare injury only when following a high energy trauma, since the hip joint is a highly stable joint. However, a much lower energy is needed for dislocation of the hip in children. The classification of traumatic hip joint dislocation into anterior and posterior dislocations was based on a dividing line that connects the anterior superior iliac spine with the ischial tuberosity. Posterior hip dislocation is the most common dislocation, which is about 9 times more frequent than the anterior type [1]. Anterior dislocation is rare. The case of anterior hip dislocation with combined ipsilateral femoral shaft fracture is extremely rare, only a few cases in children being reported in the literature [2]. Here, we report a case of traumatic anterior hip dislocation associated with bilateral femoral fractures in a 7-year-old boy. To the best of our knowledge, this is the first time such injury has been reported in the literature.

## Patient and observation

**Figure 1:**
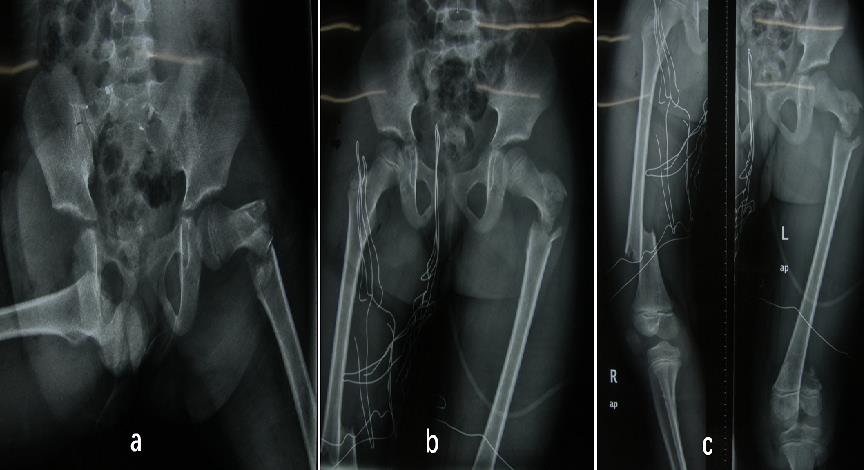
(A) the right femoral head was dislocated anteroinferiorly with a displaced contralateral proximal femoral fracture; (B) the right dislocated hip was reduced; (C) as shown in the anteroposterior (AP) view of pelvic. AP view of femurs shows the right transverse femoral shaft fracture and proximal and distal femoral bifocal fractures of the left femur

The institutional review board (The First Hospital of Jilin University) approved this work and the informed consent was obtained. A 7-year-old boy was injured in a car accident while sitting on the back passenger seat. According to the report, he was not secured in an appropriate child restraint at the time of the accident. But details of the mechanism of the injury were unknown. When admitted local hospital, his right hip joint exhibited abduction and external rotation. He had marked swelling and pain through the hip to the femur. There were soft tissue contusion and open injury of proximal thigh. In the groin area, an ovoid formation of hard consistency can be detected by palpation. X-ray findings in local hospital revealed that the right femoral head was dislocated anteroinferiorly. The ipsilateral femoral shaft showed a transverse femoral shaft fracture, and proximal and distal femoral bifocal fractures of the contralateral femur (Figure 1). The hip dislocation was reduced and debridement was performed under general anesthesia in more than 10 hours. The patient was transferred to our hospital after injured for 36 hours when his condition was stable.

**Figure 2:**
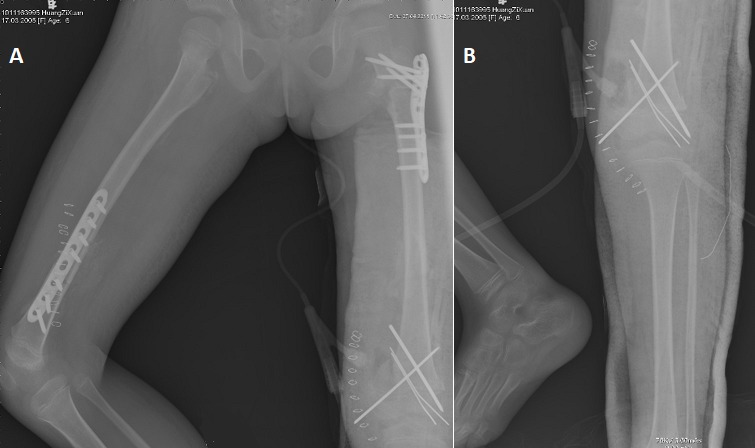
(A) the left and right femoral shaft fracture was fixed with proximal humerus internal locking system and straight plate and screws respectively (B) and the distal femoral fracture was fixed with Kirschner wires

When the patient was admitted to our emergency department, he was conscious and alert, well oriented with stable vital signs on arrival. His major complaints were pain and swelling of bilateral thigh. There were good movements of the toes with palpable dorsalis pedis artery. No signs of any peripheral neurovascular lesions existed. Hemoglobin was low, which was considered as hemorrhagic anemia caused by multiple fractures. After blood transfusion treatment, anemia was corrected. Other coexisting abdominal, thoracic, neurological or musculoskeletal lesions were excluded using clinical and laboratory examinations. After received twice of blood transfusion treatment, the patient underwent surgery 1 week after trauma. Under general anesthesia, the right femoral shaft fracture was reduced and fixed with straight plate and screws, and the left proximal femoral fracture was reduced and fixed with proximal humerus internal locking system (PHILOS^TM^, Depuy Synthes), closed reduction of the left distal femoral fracture was accomplished and held with Kirschner wires. Intra- and post-operative radiographs confirmed excellent reduction of the fragments (Figure 2). The reduced hip was noted to be stable with a full range of motion.

**Figure 3:**
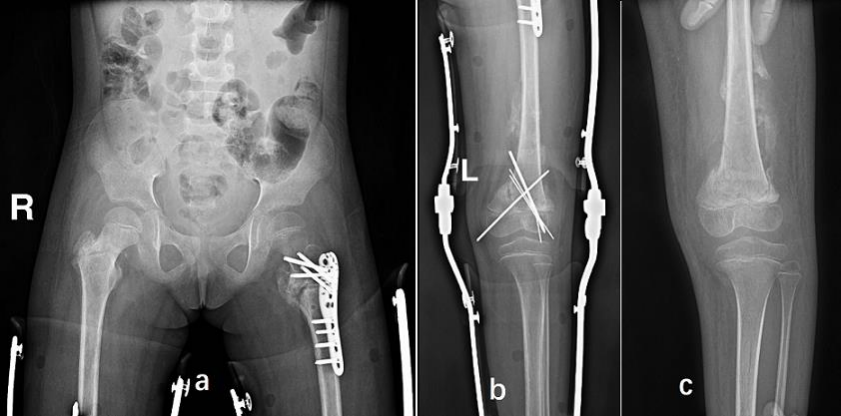
(A,B) three months after the injury, the fractures healed well as shown in the pelvic view; (C) the Kirschner wire was removed from the left distal femur

**Figure 4:**
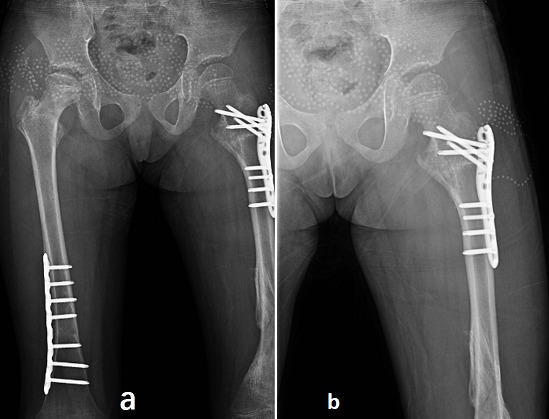
(A) six months after the injury, the fractures were healed and the shape of the right femoral head was irregular in the pelvic view; (B) the left proximal femoral fracture united and the shape of the left femoral head was normal

**Figure 5:**
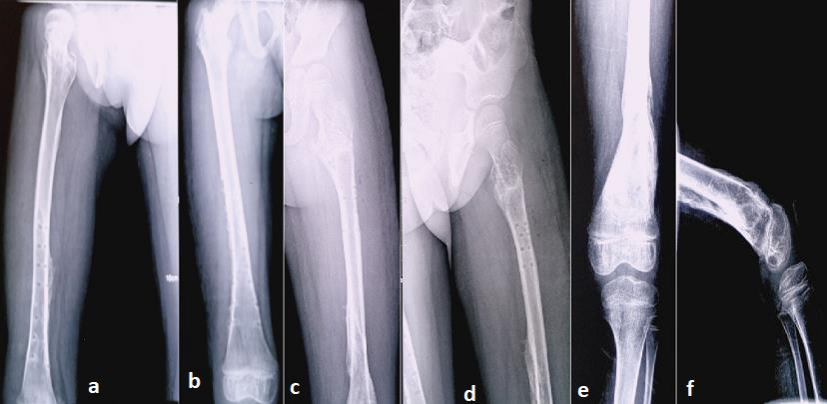
(A) eight months after the injury, anteroposterior (AP) and lateral views of right femur showed that the fractures were healed; (B) AP and lateral views of left hip and knee showed that the left proximal femoral fracture; (C) and the left distal femoral fracture were united

Postoperatively, the patient was advised to rest in bed for 3 months. Antibiotics were used during the perioperative period with Ceftazidime 400 mg daily for 2 days. Physical therapy including predominantly isometric exercises for toning the muscles, at 3 weeks hip joint mobilization exercises was started immediately after surgery. For post-operative follow-ups, X-rays examinations were performed immediately after surgery (Figure 2), at 3 months (Figure 3), at 6 months (Figure 4), at 8 months (Figure 5). At three months after the operation, the fractures were healed well and the Kirschner wire was removed from the left distal femur (Figure 3) and progressive weight bearing of the injured limb was allowed. At 6 months after the first operation, the fractures were healed and all the hardware was removed (Figure 4). At the last follow-up, radiographs showed the fractures healed well, however, irregularities of the femoral head suggested avascular necrosis (AVN) of the femoral head. The patient was free of pain with a complete range of hip and knee motion. After 8 months, the patient was lost during the follow-up due to his personal reasons.

## Discussion

Traumatic hip dislocations are uncommon injuries in children, constituting less than 5% of pediatric dislocations [3]. Earlier reports indicated that the rate of anterior hip dislocation in children was from 7.5% to 17.8% [4-8]. Traumatic anterior hip dislocation associated with ipsilateral femoral shaft fracture is a rare injury and even rarer in pediatric patients. In this case, the patient was suffered from not only the anterior hip dislocation with ipsilateral femoral shaft fracture, but also the contralateral bifocal femoral fracture though the mechanism is unknown. To the best of our knowledge, this is the first reported injury in literature. The hip is a stable spheroidal type of joint and reinforced by a thick articular capsule and strong ligaments. Children require a high-energy mechanism to dislocate the hip [9]. In our case, the child was assumed sitting in a relaxed position with the legs and hips flexed, abducted and externally rotated, after a high energy trauma. Combined external forces were applied to the right leg force on the knee joint that causes hip joint forced abduction and external rotation and dislocation and a force orthogonal to the femur shaft that causes transverse fracture [10]. The contralateral bifocal femoral fracture was assumed due to direct blow at the fracture sites. The goal in the treatment of a dislocated hip is to obtain anatomic reduction as soon as possible. Delay in reduction or neglected dislocations would result a high incidence of AVN [11-13]. Generally, closed reduction should be attempted initially. Although most hip dislocations in children can be reduced easily, incomplete reductions can occur from interposed soft tissue or bony fragments [14]. Proximal physeal separations with attempted closed reduction have been reported [15]. Therefore, fluoroscopy should be performed to ensure that physeal separation does not occur and the hip is completely reduced. Open reduction is indicated if the hip cannot be reduced or if there is a femoral head fracture or an incarcerated fragment [5, 16]. The violence of the reducing and the resistance of muscle contraction can make the femoral epiphysis separated from the femoral neck. Hence, complete muscle relaxation and the ability to urgently open the hip is often helpful and this is best provided in the operating room under anesthesia.

In the present case, the dislocation was reduced with general anesthesia and postoperative imaging showed complete reduction of the hip joint without femoral epiphysis separation. Avascular necrosis (AVN) of the femoral head is a recognized complication of hip dislocation. This complication was not only from the vascular organic lesion with the damaged joint capsule, the round ligament artery and the ligament, but also from a spasm of the cervical branch of medial circumflex femoral artery. The latter can be reversed by early reduction of the dislocated hip, and then the risk of AVN could be reduced from 40% to 10% if the reduction happens within less than 6 hours [16]. The prolong of non-weight bearing period is another method to prevent the AVN of the femoral head [16, 17]. It is clinically useful in adult cases. Studies have shown that even if MRI findings showed abnormal signals after reduction, after 3 months non-weight bearing, MRI findings were normalized or improved and AVN of the femoral head did not occur [18]. In the present case, unfortunately, the patient was not reduced until 10 hours after the injury, and the patient appeared AVN of the femoral head 6 months after surgery. Elastic intramedullary nailing should be the first choice for femoral shaft fractures in children [19]. But in this case, the soft tissue condition of the proximal part of the right femur of the patient was poor. If the method is used, the risk of infection is greater. Therefore, open reduction and internal fixation with plate and screw were performed. For the contralateral bifocal femoral fracture, two separate fractures can be considered, which were the proximal femoral fracture and the distal femoral fracture. For proximal femoral fractures, we innovatively used proximal humerus locking plates. The fracture of the distal femur was located above the epiphysis. We used a smooth Kirschner wire, which fixed the fracture well and avoided the presence of a threaded instrument that disturbs the epiphysis. At six months follow up, fractures were well healed and there was no apparent growth abnormality. Based on our experience, a few notes were summarized below: 1) Reduction of the dislocated hip is an emergency that should be addressed as soon as the patient's condition allows to avoid AVN of the femoral head. 2) For children with proximal femoral fractures, the use of philos plates produces good results. 3) Patients should be closely monitored for recurrent subluxation, dislocation and AVN.

## Conclusion

This case stresses the importance of a rapid evaluation and treatment for the hip dislocation. Early physical rehabilitation and minimized the risk of complications could be expected after a stable reduction and a firm internal fixation of the associated fractures.

## Competing interests

The authors declare no competing interests.

## References

[1] Dreinhöfer KE, Schwarzkopf SR, Haas NP, Tscherne H (1994). Isolated traumatic dislocation of the hip: Long-term results in 50 patients. Journal of Bone & Joint Surgery-British.

[2] Yamamoto K, Ko M, Masaoka T, Shishido T, Imakiire A (2004). Traumatic anterior dislocation of the hip associated with ipsilateral femoral shaft fracture in a child: a case report. Journal of Orthopaedic Surgery.

[3] Fernandez FF, Wirth T, Eberhardt O (2012). Acute traumatic and especially neglected traumatic hip dislocations are very rare in children. Unfallchirurg.

[4] Barquet A (1979). Traumatic hip dislocation in childhood: a report of 26 cases and a review of the literature. Acta Orthopaedica Scandinavica.

[5] Funk F, James JR (1962). Traumatic dislocation of the hip in children: factors influencing prognosis and treatment. The Journal of Bone and Joint Surgery.

[6] Offierski CM (1981). Traumatic dislocation of the hip in children. Journal of Bone & Joint Surgery British.

[7] Pearson DE, Mann RJ (1973). Traumatic hip dislocation in children. Clin Orthop Relat Res.

[8] Salisbury RD, Eastwood DM (2000). Traumatic dislocation of the hip in children. Clinical Orthopaedics & Related Research.

[9] Gittins ME, Serif LW (1991). Bilateral traumatic anterior/posterior dislocations of the hip joints: case report. Journal of Trauma.

[10] Malkawi H (1982). Traumatic anterior dislocation of the hip with fracture of the shaft of the ipsilateral femur in children. Journal of Pediatric Orthopaedics.

[11] Bunnell WP, Webster DA (1980). Late reduction of bilateral traumatic hip dislocations in a child. Clinical Orthopaedics & Related Research.

[12] Dhillon MS, Sharad P, Kamal B, Chouhan D, Kumar V (2011). Functional outcome of neglected perilunate dislocations treated with open reduction and internal fixation. Indian Journal of Orthopaedics.

[13] Kumar S, Jain AK (2005). Neglected traumatic hip dislocation in children. Clinical Orthopaedics & Related Research.

[14] Jaskulka RA, Fischer G, Fenzl G (1991). Dislocation and fracture-dislocation of the hip. Journal of Bone & Joint Surgery-british Volume.

[15] Herrera-Soto JA1, Price CT, Reuss BL, Riley P, Kasser JR, Beaty JH (2006). Proximal femoral epiphysiolysis during reduction of hip dislocation in adolescents. Journal of Pediatric Orthopedics.

[16] Freeman George E (1961). Traumatic Dislocation of the Hip in Children: a report of seven cases and review of the literature. J Bone Joint Surg.

[17] Glass A, Powell HDW (1961). Traumatic dislocation of the hip in children: an analysis of forty-seven patients. Reconstruction Surgery & Traumatology.

[18] Poggi JJ, Callaghan JJ, Spritzer CE, Roark T, Goldner RD (1995). Changes on magnetic resonance images after traumatic hip dislocation. Clinical Orthopaedics & Related Research.

[19] Andreacchio A, Marengo L, Canavese F, Pedretti L, Memeo A (2016). Comparison between external fixation and elastic stable intramedullary nailing for the treatment of femoral shaft fractures in children younger than 8 years of age. Journal of Pediatric Orthopaedics-part B.

